# Comparison of resident and glaucoma faculty practice patterns in the care of open-angle glaucoma

**DOI:** 10.1186/s12886-015-0027-x

**Published:** 2015-04-14

**Authors:** Nazlee Zebardast, Jason F Solus, Harry A Quigley, Divya Srikumaran, Pradeep Y Ramulu

**Affiliations:** Wilmer Eye Institute Johns Hopkins University, 600 North Wolfe St. Maumenee B-110, Baltimore, MD 21287 USA

**Keywords:** Glaucoma, Resident education, Preferred practice patterns

## Abstract

**Background:**

Previous studies suggest there are large variations in adherence of ophthalmologists with the American Academy of Ophthalmology’s Preferred Practice Patterns (PPPs). The purpose of this study was to compare rates of compliance with glaucoma care guidelines between resident and glaucoma faculty physicians at a single institution.

**Methods:**

Charts of resident continuity clinic or glaucoma faculty patients with primary open angle glaucoma (POAG), ocular hypertension (OHTN), or suspicion of glaucoma were reviewed during the 2005–6 academic year. Performance within care measures specified by the 2005 PPP guidelines was compared between resident and faculty physicians using univariate and multivariable logistic regression models.

**Results:**

112 resident and 100 faculty charts were reviewed. The mean compliance rate for all 7 care measures for resident physicians was significantly lower than that of faculty physicians (78% vs. 96%, p < 0.001). As compared to glaucoma faculty, resident physicians were less likely to have documented 6 of the 7 individual care measures (p ≤ 0.001 for all); the exception was optic nerve (ON) description. In multivariable analyses, resident patients were more likely to have at least one undocumented care measure than faculty patients (OR = 10.1, 95% CI = 5.1 to 20.0, p < 0.001). Among resident patients, undocumented care measures were more common among patients with poorer visual acuity (VA) in the better eye.

**Conclusions:**

Though unmeasured differences in clinic structure and patient characteristics may have partially contributed to poorer resident performance, residents were more likely than faculty to omit PPP care measures and significantly underperformed faculty in global assessment of glaucoma care. Resident education should focus on integration of PPPs into residency training and monitoring of resident compliance with evidence-based guidelines.

## Background

Glaucoma is the second leading cause of blindness worldwide [1], and treatment of glaucoma accounts for a significant portion of ophthalmology visits in the United States (US). As of 2011, over 2.7 million individuals are estimated to have primary open angle glaucoma (POAG) in the US [2], a number projected to increase to 7.3 million by 2050. Given the prevalence of glaucoma in the US population, the early detection and proper treatment of glaucoma is critical for preventing vision loss. The American Academy of Ophthalmology (AAO) has devised treatment guidelines called Preferred Practice Patterns (PPPs) for many ophthalmologic diseases, including glaucoma [3]. These PPPs are designed to standardize clinical practices and to ensure quality medical care through the promulgation of evidence-based criteria for frequency of examinations, required history-taking, and specific evaluation techniques.

Previous reports have suggested that adherence to PPPs by fully trained ophthalmologists for various eye pathologies [4-6], including glaucoma [7-11], vary widely and may be less than ideal. In glaucoma management, while there is good compliance with documentation of intraocular pressure (IOP), clinical optic disc evaluation [7,8,10,11], and follow-up visit frequency [8] for patients with stable glaucoma, deviations from the recommended guidelines are common with regards to performing gonioscopy [8,10,11], measuring central corneal thickness (CCT) [11], setting a target IOP [8,11], obtaining visual fields (VFs) [8,9], and obtaining regular glaucoma imaging [9,11].

Integrating evidence-based guidelines such as the PPP during ophthalmology resident training may help establish appropriate habits of clinical care. Assessment of resident compliance with the PPP [12-16] may provide an objective method to achieve the mandate of the Accreditation Council for Graduate Medical Education (ACGME) to “provide objective assessments of competence in patient care and procedural skills, medical knowledge, and practice-based learning and improvement” [17]. In addition, payers, such as Medicare, are now using practice-based measures to evaluate the quality of care.

Three previous studies have examined the compliance of resident physicians with PPP guidelines for cataract [18], diabetic retinopathy (DR) [19], and POAG [20]. Overall compliance rates varied considerably from 52% for initial diabetic eye examination, 81% for cataract operative and postoperative management, and 83% for POAG follow-up examination. None of these studies compared resident physician performance to that of senior ophthalmologists at the same institution. Here, we compare adherence with the glaucoma PPP standards for residents and faculty at a single institution.

## Methods

The study protocol was approved by the Johns Hopkins Institutional Review Board (IRB). Consent was obtained from all faculty participants. With IRB permission, waivers of consent were obtained for all patients whose charts were examined and for resident participants, obviating the need for signed consent from these groups.

### Participating residents and faculty

Charts were evaluated from patients visiting the resident-run general ophthalmology continuity clinic of 14 Johns Hopkins ophthalmology residents in their second or third year of ophthalmology training, and from patients visiting the glaucoma subspecialty clinics of 5 full-time glaucoma-division faculty members, during the 2005–6 academic year. Glaucoma faculty and a fellowship-trained chief resident were available for assistance in care of patients seen at the resident-run general ophthalmology clinic upon residents’ request, but were not required to see any specific set of patients or to review patient charts. Based on historical trends, on average, residents saw approximately 25 patients per day whereas glaucoma faculty saw approximately 50 patients per day. Technical staff was available for all residents and faculty members though the exact ratios of technician to physician and technician patient load are not known. Faculty physicians, but not resident physicians, used an electronic medical record system at the time of this study (data did not auto-populate in this system). Neither faculty nor resident physicians used scribes or other physician extenders.

Consecutive charts, beginning at the start date of the study period (January 2006), were reviewed by a senior faculty member or by an individual (JFS) trained in chart abstraction and with documented error rate in any care measure of <10%. Twenty charts were analyzed for each glaucoma faculty member, while 8 patient charts were analyzed for each resident physician. Residents began caring for glaucoma patients in their first year of ophthalmology training and therefore had at least 18 months of experience in the care of such patients at the time of the study.

### Study sample

Patients of the participating faculty and residents were identified using billing and/or visit records. A preliminary chart review was conducted to assess whether patients satisfied the entry/exclusion criteria. Included patients had a diagnosis of POAG, open angle glaucoma suspect, or ocular hypertension (OHTN). Exclusion criteria included: (1) age less than 18, (2) diagnosis of secondary glaucoma (e.g. traumatic, uveitic or neovascular glaucoma), (3) diagnosis of angle closure glaucoma, and (4) a most recent visit within the global post-operative period for any ophthalmic procedure.

### Study period

All analyzed charts had a visit occurring between January 2006 and June 2006, and at least 2 additional visits prior to this date at our institution. Additional visits following the first visit within the January 2006 – June 2006 time period were not analyzed.

### Data extraction

Data collected from each patient chart included demographic details, visual acuity (VA) and IOP in each eye, current IOP-lowering medications, history of previous laser or surgical treatment for glaucoma, and details of the most recent VF. We assessed 7 distinct care measures that are included in the 2005 AAO PPP for POAG [21]. The entire chart was reviewed for every patient and credit was given if the data were documented at any location within the chart for the following 4 criteria: gonioscopy, CCT estimation, specific setting of target IOP, and recording of family history of glaucoma. The clinical description of optic disc and presence of VF testing in the chart were given credit if performed within the last 12 months. The seventh criterion was the presence of photographic (stereoscopic and/or non-stereoscopic) or digital glaucoma imaging (i.e. computer-based imaging of the optic nerve head and/or retinal nerve fiber layer) in the 5 years prior to the study visit. Any photographic/digital imaging of the optic nerve and/or retinal fiber layer (or detailed drawing of the optic nerve head in the absence of imaging technologies) was combined into one category, as per PPP classification. Target IOP was employed as a care measure only in patients undergoing treatment. Patients were considered under treatment if they were currently on IOP-lowering medications or had a history of previous laser or surgical treatment to lower IOP. Credit for all care measures was given if the measure was performed in at least one eye.

### Statistical analysis

Analyses were conducted using STATA 12.0 (College Station, Texas). Group differences in continuous variables were evaluated by Student’s t-test, while differences in categorical variables were evaluated with the chi-squared test.

Mean compliance and performance within single care measures were compared between resident and faculty physicians using univariate analysis. Mean compliance was taken as the average performance for all 7 care measures. Multivariable logistic regression models were then used to assess the odds ratio (OR) of omitting each individual care measure by faculty and resident groups. For each patient chart reviewed, a composite score was calculated by assigning a single credit for each performed care measure for a total of 6 or 7 points depending on whether the patient was considered under treatment. Using the composite score we assessed the odds of omitting at least one care measure by faculty and resident groups in multivariable models. Patient age, diagnosis, and better eye VA were employed as covariates in all multivariable models. Patient characteristics predicting performance of each care measure by resident and faculty physicians were also evaluated using univariate logistic regression models. Using the methodology detailed above, we then compared the performance of care measures between residents in their second and third year of training.

## Results

Charts were reviewed for 212 patients, including 112 cared for by resident physicians and 100 cared for by glaucoma faculty. Patients cared for by resident and faculty physicians were not significantly different in age, gender, glaucoma diagnosis, medication usage, IOP, VF mean deviation or VA (Table 1). Patients cared for by residents were more likely to be African American (85% vs. 26%, p < 0.001).

**Table 1:** **Characteristics of patients cared for by resident and faculty physicians**

**Patient characteristic**	**Residents (n = 112)**	**Faculty (n = 100)**	**p**
Age in years, mean (SD)	68.3 (12.3)	67.1 (11.8)	0.49
Female gender, %	58.9	50.0	0.19
African American, %	84.8	26.0	<0.001
Glaucoma suspect, %	39.3	31.0	0.21
Glaucoma medications used, mean (SD)	1.38 (1.3)	1.51 (1.2)	0.44
IOP, right eye in mm Hg, mean (SD)	17.7 (8.9)	15.9 (4.2)	0.06
IOP, left eye in mm Hg, mean (SD)	17.8 (7.7)	17.0 (5.3)	0.43
Better eye VF MD, mean (SD)	−5.7 (9.1)	−3.6 (5.6)	0.06
Worse eye VF MD, mean (SD)	−7.7 (9.3)	−7.7 (7.6)	0.98
Better eye VA in logMAR, mean (SD)	0.21 (0.48)	0.12 (0.26)	0.11
Worse eye VA in logMAR, mean (SD)	0.71 (1.02)	0.48 (0.78)	0.08

SD = standard deviation; IOP = intraocular pressure; mm Hg = millimeters of mercury; VF = visual field; MD = mean deviation; VA = visual acuity; logMAR = logarithm of the minimum angle of resolution.

Overall, the mean compliance for all 7 care measures for resident physicians was 78% compared with 96% for faculty physicians (p < 0.001). As compared to resident charts (Table 2), faculty charts were more likely to have a documented gonioscopic exam (98% vs. 76.8%, p < 0.001), more likely to have recorded CCT (100% vs. 63.4%, p < 0.001) and more likely to record family history of glaucoma (100% vs. 87.5%, p < 0.001). Faculty patients were also more likely to have undergone VF testing in the past year (96% vs. 80.4%, p = 0.001) and to have had glaucoma imaging in the previous 5 years (87% vs. 61.6%, p < 0.001). Among patients receiving treatment, residents documented a target IOP in 73.8% of patients, while faculty charts did so in 100% of cases (p < 0.001). While residents were more likely to omit any of the other measures, they were more likely than faculty to have documented ON description upon clinical exam (100% vs. 94%, p = 0.009). Overall, residents and faculty omitted an average of 1.5 (95% CI = 1.2 to 1.8) and 0.25 (95% CI = 0.2 to 0.3) measures respectively (p < 0.001). Moreover, 42% of patients treated by residents had two or more undocumented care measures, compared with 2% for faculty treated patients (p < 0.001). There were no significant differences in both mean compliance and performance on each individual care measure between residents in their second and third year of training (p > 0.05 for all measures).

**Table 2:** **Variations in documentation of care measures by resident status, univariate analysis**

**Care measure**	**Resident**	**Faculty**	**p**
Gonioscopy (%)	76.8	98.0	<0.001
CCT recorded (%)	63.4	100.0	<0.001
Target IOP recorded (%)	73.8	100.0	<0.001
FHx recorded (%)	87.5	100.0	<0.001
VF in last 12 months (%)	80.4	96.0	0.001
ON Description, last 12 mos. (%)	100.0	94.0	0.009
Glaucoma imaging, last 5 yrs (%)	61.6	87.0	<0.001

CCT = central corneal thickness; IOP = intraocular pressure; FHx = family history; VF = visual field; ON = optic nerve;

p values test significance of differences between resident and faculty physicians.

Differences in documentation of care measures by faculty and residents were assessed in multivariable analyses adjusting for age, stage of disease (suspect/OHTN vs. glaucoma), and better-eye VA. Overall, resident patients were more likely to have at least one undocumented care measure than faculty patients (OR = 10.1, 95% CI = 5.1 to 20.0, p < 0.001). With regard to individual quality measures, resident patients were more likely to have undocumented gonioscopic exam (OR = 13.2, 95% CI = 3.0 to 58.5, p = 0.001), to have not had a VF test in the last 12 months (OR = 5.5, 95% CI = 1.7 to 18.0, p = 0.004), and to have not undergone glaucoma imaging in the last 5 years (OR = 4.1, 95% CI = 2.0 to 8.5; p < 0.001), as compared to faculty patients. Large differences were also found between resident and faculty patients with regards to documentation of glaucoma family history, CCT, and target IOP, though the significance of these differences could not be determined in multivariable logistic regression models as no failures were observed amongst faculty patients.

In both faculty and resident patient groups, we separately assessed the effect of several patient parameters on documentation of care measures using univariable analyses (Table 3). Among faculty patients, those with more advanced VF loss in the better eye at last testing were more likely to have had VF testing in the last year (odds of missed VF testing = 0.3 per 5 dB worse VF mean deviation, 95% CI = 0.2 to 0.7, p = 0.003). In addition, female patients were less likely to have had glaucoma imaging in the past 5 years (OR = 3.9 when compared to male patients, 95% CI = 1.0 to 15.2, p = 0.049).

**Table 3:** **Patient characteristics predicting performance of care measures in individuals treated by resident physicians**

		**No documented gonioscopy**	**No CCT documentation**	**No target IOP recorded**	**No Fam Hx documented**	**No VF in last year**	**No glaucoma imaging in last 5 years**
**Patient characteristic**	**Interval**	**Odds ratio**	**Odds ratio**	**Odds ratio**	**Odds ratio**	**Odds ratio**	**Odds ratio**
Age	5 years older	1.18	1.20*	0.93	1.27	1.06	0.94
Gender	Female vs. Male	0.76	0.97	0.91	0.92	1.27	0.70
Race	AA vs. not AA	5.71	1.07	0.76	2.54	4.54	0.87
Diagnosis	Suspect vs. Glaucoma	0.77	1.35	0.60	0.22	0.51	0.53
Glaucoma medicines	1 more medication	1.23	1.08	0.65	1.13	1.06	1.53**
VF MD	5 dB worse in better eye	0.91	0.92	1.06	0.68**	1.13	0.86
Visual acuity, logMAR	0.1 worse in better eye	1.14**	1.19*	1.11*	1.14**	1.17**	1.08

CCT = central corneal thickness; IOP = intraocular pressure; Fam Hx = Family History; VF = visual field; AA = African American; MD = mean deviation; dB = decibel; logMAR = logarithm of the minimum angle of resolution;

p values test the importance of each patient characteristic on the given outcomes (care measures) within resident-treated patients, *p < 0.05, **p < 0.01.

Among resident patients, undocumented gonioscopy was more common among patients with poorer VA in the better eye (OR = 1.1 per 0.1 logMAR decrement in VA, 95% CI = 1.0 to 1.3, p = 0.009), while undocumented CCT was more common among older patients (OR = 1.2 per 5 years of age, 95% CI = 1.0 to 1.4, p = 0.033) and those with poorer VA in the better eye (OR = 1.2 per 0.1 logMAR decrement in VA, 95% CI = 1.0 to 1.4, p = 0.012). Among patients receiving treatment, undocumented target IOP was more common in patients with poorer better eye VA (OR = 1.1 per 0.1 logMAR decrement in VA, 95% CI = 1.0 to 1.2, p = 0.027). Undocumented family history of glaucoma was more common among patients with poorer better eye VA (OR = 1.1 per 0.1 logMAR decrement in VA, 95% CI = 1.0 to 1.3, p = 0.006). However, patients with more advanced VF loss in the better eye were more likely to have documentation of glaucoma family history (odds of missed VF testing = 0.7 per 5 dB worse VF mean deviation, 95% CI = 0.5 to 0.9, p = 0.008). In addition, patients with poorer better eye VA were more likely to have not undergone VF testing in the last year (OR = 1.2 per 0.1 logMAR decrement in VA, 95% CI =1.0 to 1.3, p = 0.004), while patients on one or more glaucoma medications were more likely to lack glaucoma imaging in the past 5 years (OR = 1.5 per one more medication, 95% CI = 1.1 to 2.1, p = 0.006).

## Discussion

The selected key elements of the PPP guidelines for glaucoma care were documented differently between glaucoma faculty and residents at our institution. The overall compliance rate for resident physicians was significantly lower than that of faculty physicians. In univariate analyses, residents had a greater number of undocumented care measures and underperformed faculty in 6 of the 7 individual care measures assessed. In multivariable models, residents had 10-fold higher odds of failing to document at least one care measure as compared to faculty. These findings suggest that resident care at our institution failed to achieve the standard set by faculty performance. Among faculty patients, adherence to all measures of glaucoma care was high; there were no omissions in 3 of 7 care measures and only 2% of faculty-treated patients had two or more undocumented care measures. The adherence of faculty to guidelines for glaucoma care in the present study was higher than rates previously reported for gonioscopy (46-51%) [8,10,11], CCT (52%) [11] and setting of a target IOP (1-19%) [8,11] by community ophthalmologists. The rate of gonioscopy for our study is comparable to the 85% previously reported for an initial visit at an academic glaucoma referral center [7].

The frequency of our faculty patients having a timely VF (96%) was similar to rates reported for community-based ophthalmologists (90%) [10,11] and for academic-based glaucoma specialists (92%) [7], and was substantially higher than that for studies using claims data to identify performed care (46%-66%) [8,9]. The latter approach may underestimate actual evaluation rates. Faculty adherence to clinical description and evaluation of the optic disc was largely in accordance with previous studies (90-98%) [7-11]. However, reported glaucoma imaging rates in previous studies using claims data were lower than that found in the present study. These rates vary widely from 13% [9] for 1995–1999 to 78% [11] for 2001–2004 study periods. The higher rates of glaucoma imaging observed in the latter study and in our study may reflect increased use and acceptance of digital imaging technologies. Interestingly, faculty patients who were female were less likely to have glaucoma imaging. The reasons for this finding are not completely clear and certainly warrant further investigation to determine if there is indeed a true gender bias.

It is difficult to compare the glaucoma PPP compliance rates for resident physicians at our institution to studies that focused on fully-trained community ophthalmologists [7-11]. One recent study reported compliance rates of 93% for ON and VF examinations by first year and second year residents on follow-up visits, which is comparable to the present study [20]. Interestingly, we also found that the poorer better-eye VA was associated with failure to comply with nearly all of care measures among resident physicians. This may be due to inherent difficulty and decreased reliability of diagnostic tests, such as automated perimetry, in advanced stages of glaucoma [22,23]. Yet the same association was not observed for patients treated by faculty physicians. Perhaps the longer-term relationship of faculty physicians with advanced disease patients, combined with a possible lack of care continuity on a resident service, contributes to less than optimal glaucoma management in such patients.

In addition to greater experience and continuity of care provided by faculty, other possible interpretations for why residents fell short in ideal documentation of care should be considered. First, resident physicians may not yet be fully comfortable with performing procedures such as gonioscopy or may not have been as cognizant as faculty of the PPP guidelines. Second, it is possible that the measured rates of compliance with respect to imaging and VF tests may have been lower in the resident group due to instrument/technician availability (resident clinic patients typically had to use imaging from the faculty clinic which may have provided a barrier to these tests), differences in the rates of missed visits, or unmeasured differences in the types of patients treated. For example, residents may have been caring for higher percentage of uninsured patients, resulting in an attempt to conserve resources by limiting VF testing and obtaining glaucoma imaging when possible. In addition, there was a significant difference in the racial distribution of patients treated by faculty and resident physicians, and patients of different race may receive different treatments because of physician behavior and/or patient acceptance of testing. However, it should be noted that race did not predict differences in documentation of care measures in either resident or faculty patients. Third, faculty, but not residents, at our institution utilized an electronic data entry system during the study period which had locations for documentation of each care measure. While it was possible to omit such findings and complete the note, the presence of cues to document the care measure may be associated with better adherence with guidelines. Finally, it is possible that examinations were performed by residents, but not documented at the same rate as by faculty. The latter would be of concern as explicit documentation of care processes is particularly important for resident physicians to allow for consistent care across providers over time.

Though there are differences in methodology with prior studies, our residents’ overall compliance rate of 78% with global glaucoma PPP guidelines is comparable to that reported for POAG follow-up evaluation (83%) [20] and cataract surgical management and postoperative evaluation (81%) [18], and higher than that reported for residents’ initial evaluation of DR (52%) [19]. These large variations in performance by resident physicians suggest there is a need to develop standardized evaluation tools to assess residents’ performance. The incorporation of PPPs into residency training can enhance resident education and may help eliminate the discrepancies in practices between resident and faculty physicians. In addition, measuring adherence to the PPP provides a useful and objective measure of resident performance, personal clinical efficacy, and identifies potential areas for improvement. Given the continuing advances in medicine, there is also a need to periodically re-monitor resident compliance with practice patterns as standards of care change over time. Previous studies, which explored the incorporation of PPPs into residency training demonstrated that most residents are not adequately familiar with the evidence-based standards of care in their fields, but view PPPs as helpful in guiding clinical decision-making [12-16]. Measurement of resident practice patterns would also complement subjective written evaluations by faculty and better fulfill the ACGME’s mandate for providing “objective assessments of competence in patient care and procedural skills, medical knowledge, and practice-based learning and improvement” [17]. However, it is important to note that the PPPs serve as guidelines and may not always be ideal. For example, while the PPP recommends noting family history in POAG patients [21], there is no evidence that family history is a risk factor for progression in those with known glaucoma (though it would be important to tell patients to have their family members evaluated for glaucoma).

Our study is limited in that we only examined practice patterns at one academic institution and as such we cannot draw conclusions about general resident and faculty practice patterns. The retrospective study design limited our ability to measure potentially important covariates. In addition, only the adherence to documentation of care measures was evaluated and was not related to clinical outcomes, such as disease progression and/or visit or medication compliance, though studies from other fields of medicine have shown that adherence to evidence-based guidelines may lead to improved patient outcomes [24]. Future efforts in glaucoma education and care should focus on developing care measures that not only assess whether tests are performed, but if they are interpreted correctly and are used to create effective treatment plans which are communicated appropriately to patients. Also, as most glaucoma patients at our institution are cared for in subspecialty clinics, we were unable to compare resident care of glaucoma patients to glaucoma patient care in a comprehensive ophthalmology setting where other co-existing ocular disease may be more likely.

## Conclusions

In conclusion, residents at the studied institution were much more likely than faculty to omit documentation of care measures though unmeasured differences in instrument/technician availability and patient characteristics may have partially contributed to poorer residents performance. Residents recorded important diagnostic measures over 75% of the time, however they were more likely to omit documentation of gonioscopy, measurement of CCT, setting of a target IOP and perform glaucoma imaging, at frequencies suggested by PPP guidelines. Education efforts should focus on improving resident compliance with evidence-based guidelines.

## Abbreviations

ACGME: Accreditation Council for Graduate Medical Education
AAO: American Academy of Ophthalmology
CCT: Central corneal thickness
CI: Confidence interval
dB: Decibels
DR: Diabetic retinopathy
IOP: Intraocular pressure
logMAR: Logarithm of the minimal angle of resolution
OHTN: Ocular hypertension
ON: Optic nerve
OR: Odds ratio
POAG: Primary open angle glaucoma
PPP: Preferred Practice Patterns
US: United States
VA: Visual acuity
VF: Visual field
